# Homicide rates are spatially associated with built environment and socio-economic factors: a study in the neighbourhoods of Toronto, Canada

**DOI:** 10.1186/s12889-022-13807-4

**Published:** 2022-08-04

**Authors:** Alireza Mohammadi, Robert Bergquist, Ghasem Fathi, Elahe Pishgar, Silas Nogueira de Melo, Ayyoob Sharifi, Behzad Kiani

**Affiliations:** 1 Department of Geography and Urban Planning, Faculty of Social Sciences, University of MohagheghArdabili, Ardabil, Iran; 2 Ingerod, Brastad, SE-454 94 Sweden. Formerly UNICEF/UNDP/World Bank/WHO Special Programme for Research and Training in Tropical Diseases (TDR), World Health Organization, Geneva, Switzerland; 3 Department of Human Geography, Faculty of Earth Sciences, Shahid Beheshti University, Tehran, Iran; 4 Department of Geography, State University of Maranhão, CidadeUniversitária Paulo VI, São Luís, 65055-000 Brazil; 5 Graduate School of Humanities and Social Sciences, and Network for Education and Research on Peace and Sustainability, Hiroshima University, Higashi-Hiroshima, 739-8530 Japan; 6 Centre de Recherche en Santé Publique, Université de Montréal, 7101, Avenue du Parc, Montréal, Canada

**Keywords:** Spatio-temporal analysis, Homicide, Socio-economic, Built environment, Toronto, Canada

## Abstract

**Objectives:**

Homicide rate is associated with a large variety of factors and therefore unevenly distributed over time and space. This study aims to explore homicide patterns and their spatial associations with different socioeconomic and built-environment conditions in 140 neighbourhoods of the city of Toronto, Canada.

**Methods:**

A homicide dataset covering the years 2012 to 2021 and neighbourhood-based indicators were analysed using spatial techniques such as Kernel Density Estimation, Global/Local Moran’s *I* and Kulldorff’s SatScan spatio-temporal methodology. Geographically weighted regression (GWR) and multi-scale GWR (MGWR) were used to analyse the spatially varying correlations between the homicide rate and independent variables. The latter was particularly suitable for manifested spatial variations between explanatory variables and the homicide rate and it also identified spatial non-stationarities in this connection.

**Results:**

The adjusted R^2^ of the MGWR was 0.53, representing a 4.35 and 3.74% increase from that in the linear regression and GWR models, respectively. Spatial and spatio-temporal high-risk areas were found to be significantly clustered in downtown and the north-western parts of the city. Some variables (e.g., the population density, material deprivation, the density of commercial establishments and the density of large buildings) were significantly associated with the homicide rate in different spatial ways.

**Conclusion:**

The findings of this study showed that homicide rates were clustered over time and space in certain areas of the city. Socioeconomic and the built environment characteristics of some neighbourhoods were found to be associated with high homicide rates but these factors were different for each neighbourhood.

**Supplementary Information:**

The online version contains supplementary material available at 10.1186/s12889-022-13807-4.

## Introduction

Homicide is a global public health issue [1]. The rates of homicide, one of the most severe types of violent crime, are considered around the world as a benchmark to assess the level of violent activity [1–3]. For example, Canada’s homicide rate increased from 1.83 per 100,000 population in 2019 to 1.95 per 100,000 population in 2020, which indicates a 7% increase of violence for that year [4]. The metropolitan city of Toronto, located in southern Ontario, Canada, is rapidly approaching the status of megacity [5]. The levels of violent crime and homicide are both high in many of Toronto’s neighbourhoods [6, 7]. Its police service [8] has reported that the annual number of homicides in the city increased from 57 in 2012 to 84 in 2021, with 105 homicides in 2020; the metropolitan area had the highest homicide level in Canada [9, 10]. As security is an important component of achieving sustainable and healthy cities [11, 12], high crime rates amount to a strong threat to the health of local communities [13–16]. In fact, no city can be regarded as sustainable and healthy if the occupants in their neighbourhoods lack safety [17].

The association between violent crime and communities has long been a focal point of criminological and sociological investigation [18]. High violent crime rates in cities are associated with various individual, socio-economic and environmental factors [19, 20]. The areas with less educated people are associated with more ‘criminogenic’ compared to those with higher education; specifically, areas with a low rate of people with high-school diplomas are more likely to also have many formerly incarcerated people [21, 22]. Other individual factors associated with the occurrence of violent crimes are age and gender [23]. Individuals in the 15 to 30 years age group, males in particular, run a higher risk of being involved in violent crimes [24, 25]. Socio-economic and environmental factors add to space-time clusters of violent crimes, such as homicide incidents, in urban areas that often are unevenly distributed over space and time [26–28]. Characteristics of urban neighbourhoods environment may associate with some of these spatial variations [29]; hence identifying factors that correlate with more crime in urban neighbourhoods is a central focus of this research [7, 21, 23, 24, 30, 31]. For example, socioeconomic and demographic characteristics, such as poverty, residential mobility and ethnic heterogeneity within a neighbourhood, are strongly associated with above average levels of violent crimes and urban security [15, 32, 33]. According to social disorganization theory [34], the occurrence of crime is correlated with socioeconomic and demographic variables indicating lack of cohesion, e.g., family disruption [30, 35]. For example, a neighbourhood with proportionally more poor, unemployed and low-income residents is more likely to have a higher crime rate than other neighbourhoods [36, 37]. Some studies confirm a significant correlation between median household income inequality and rental housing rate on the one hand and the rate of violent crimes on the other [32, 37]. According to Lens [38], the general incidence of violent crimes among tenant households is higher than that among homeowners, and the results of Lam's research in Toronto [39] show that homicide among minorities and new immigrants is higher than that in the majority groups. Other research has identified population density and economic activity as associated factors with high crime rates in some urban neighbourhoods [40]. Further, studies have found that built-environment characteristics, such as commercial establishments, sports places, places of interest, poor housing situations (large poorly designed buildings) and road intersections, are associated with increased homicide rates in urban areas [41–45].For example, in New York City most homicides occur in areas where many neighbourhoods intersect [44]. Further, the concentration of secondary schools in particular areas has been reported as one of many important factors correlating with increased rates of violent crime [19, 46–48].

This study pursued two main objectives. First, it attempted to identify and analyse spatial and temporal patterns of homicide rates in Toronto during 2012-2021 at the level of 140 Toronto neighbourhoods. Second, it focused on exploring the correlation(s) between the level of homicide rates on the one hand and economic, social and built environment factors on the other.

## Methodological literature review

In recent years, the fields of crime analysis, crime mapping, and environmental criminology have grown in prominence [49–52]. As a result, numerous analytical studies have been conducted with regard to various types of crimes [53, 54]. For years, spatial analysis of homicide rates has also come to the attention of crime analysts [55]. In this study, we focused on research on homicides in recent years, examining the spatial aspects of homicides in association with specific social economic and built environmental circumstances.

Graifand Sampson [56], studied the association between immigration and diversity with the homicide rate in Chicago using geographically weighted regression (GWR). They found that the association of neighbourhood characteristics with the homicide rate varied across the city, indicating a process of “spatial heterogeneity” and that immigrant concentration is either unrelated or inversely related to homicide. The GWR is commonly used to determine the spatial association among explanatory variables. Thompson & Gartner [7] used ordinary least squares (OLS) methodology and negative binomial models to explore the association between neighbourhood characteristics and homicide rates in the city of Toronto finding higher rates of violent crime and homicides in neighbourhoods where the ratios of youth and black people were higher and where the average household incomes were lower. The OLS method was used to find the best linear fit among socio-economic factors and homicide rates; however, as the explanatory factors manifested spatial variations among different neighbourhoods, the researchers suggested using a GWR model to take into account the spatial phenomena in future research [7]. A Brazilian study [57] reported higher homicide rates in communities where the majority were poor blacks with low life expectancy; using the generalized incremental regression model based on time series analysis and spatio-temporal approach they revealed an increase in homicide rate from 2000 to 2016 in the black society. Wang & Williams [30] analysed violent crimes in Toronto’s 140 neighbourhoods considering the individual factors of offenders and four dimensions of the Ontario-Marginalization Index using OLS and GWR models, showing that violent crimes were clustered in the central areas of the city. Instability and deprivation indices were used to associate with high rates of homicides in high-risk neighbourhoods. Ingram & Marchesini [58], in their analysis of homicide in Brazilian cities using geographical information system (GIS) and crime mapping, concluded that homicide occurred mainly in poor and overcrowded neighbourhoods with high unemployment rates and poor housing conditions. They also found that violent crime rates were high in neighbourhoods with high ethnic and minority diversity. The GWR-SL approach provided a framework to add unpredictable spatial interference variables to spatial variables [58].

A study in Kentucky, USA, examined the homicides rates at the county level and showed that homicide rates were higher in areas with high alcohol sales. The multilevel logistic regression was performed using clustered and non-clustered homicide areas as the binomial dependent variable; however, if the researchers had used the GWR method, the spatial association between homicide rates and independent variables would have been obtained [59]. Another study based on GIS and spatial analysis [60] showed that violent crimes and homicide rates were higher in areas where secondary schools and sport places were concentrated. Due to the scattering and excessive fragmentation of data, the negative binomial regression method was used to investigate the spatial association between homicide rates and the explanatory variables [60]. In a similar study conducted by de Miranda & de Figueiredo [43], homicide rates were higher in neighbourhoods where crowded and large buildings were concentrated and where most residents were tenants. The spatial autocorrelation methods, including hotspot analysis and Local Moran's *I* were used to identify the area where both homicide rate and at least one explanatory variable formed hotspots. Onifade [61], studied the associations between green-space areas and street crimes in Toronto concluding that violent crimes were more prevalent in areas where the density of road intersection was higher. The spatially weighted regression used in the study helped the researchers model the space-affected associations to obtain reliable results. South et al. [62], performed GWR to examine the association between structural housing repairs for low-income homeowners with neighbourhood crime in Philadelphia City, PA, USA. Here, major repair rates in low-income households were significantly associated with higher homicide and violent crime rates echoing the results of a Brazilian study using a spatial autocorrelation method conducted in João Pessoa/Paraíba [63] where the spatial patterns of intentional homicides were shown to be higher in poor districts compared to others.

Most previous studies have attempted to examine the association between homicide incidents and a specific type of variable, such as individual [7, 21, 31, 58], socio-demography [24, 30, 35, 40], economy [30, 36, 37] or built environment [43, 60, 61], separately. However, given the number of indicators available, the present study represents an attempt to assess the association between the all the different socioeconomic and built environment factors on the one hand and homicides on the other. This was done, since we feel that a comprehensive analysis of the role of each indicator can be determined more accurately by considering a large number of potential factors together. Furthermore, in terms of analytical approach, previous research studied homicide from a purely spatial aspect [6, 7, 19, 28, 30, 64] or a purely temporal one [27, 35, 39]. In this study, homicide data have been analysed from temporal, spatial and spatio-temporal point of view. The fundamental hypothesis is that the ‘where and when’ crimes are committed are not random but follow a clustered pattern [65, 66] concentrated on a small proportion of places [67]. Therefore, GIS provides a powerful tool to identify existing patterns of crimes and their spatio-temporal patterns (high-risk areas), something which is essential for the development of strategies for reducing crime [68–72]. It also assists criminal justice in improving law enforcement and implementing social and economic measures to reduce and prevent various types of crime [64, 73, 74]. Finally, previous studies rely more on traditional statistical analysis such as regression analysis [37, 40, 69] and only a few studies examined local variations or spatio-temporal patterns of homicides using location-integrated statistical analysis such as multiple GWR (MGWR) and Kulldorff’s space-time methodologies. The literature has acknowledged the neighbourhood as an appropriate scale for spatial analysis of crime incidents and useful for the determination of the association between crime rates and socio-economic and built-environmental variables [30, 47, 48, 75].

## Research methodology

### Study area

**Fig. 1:**
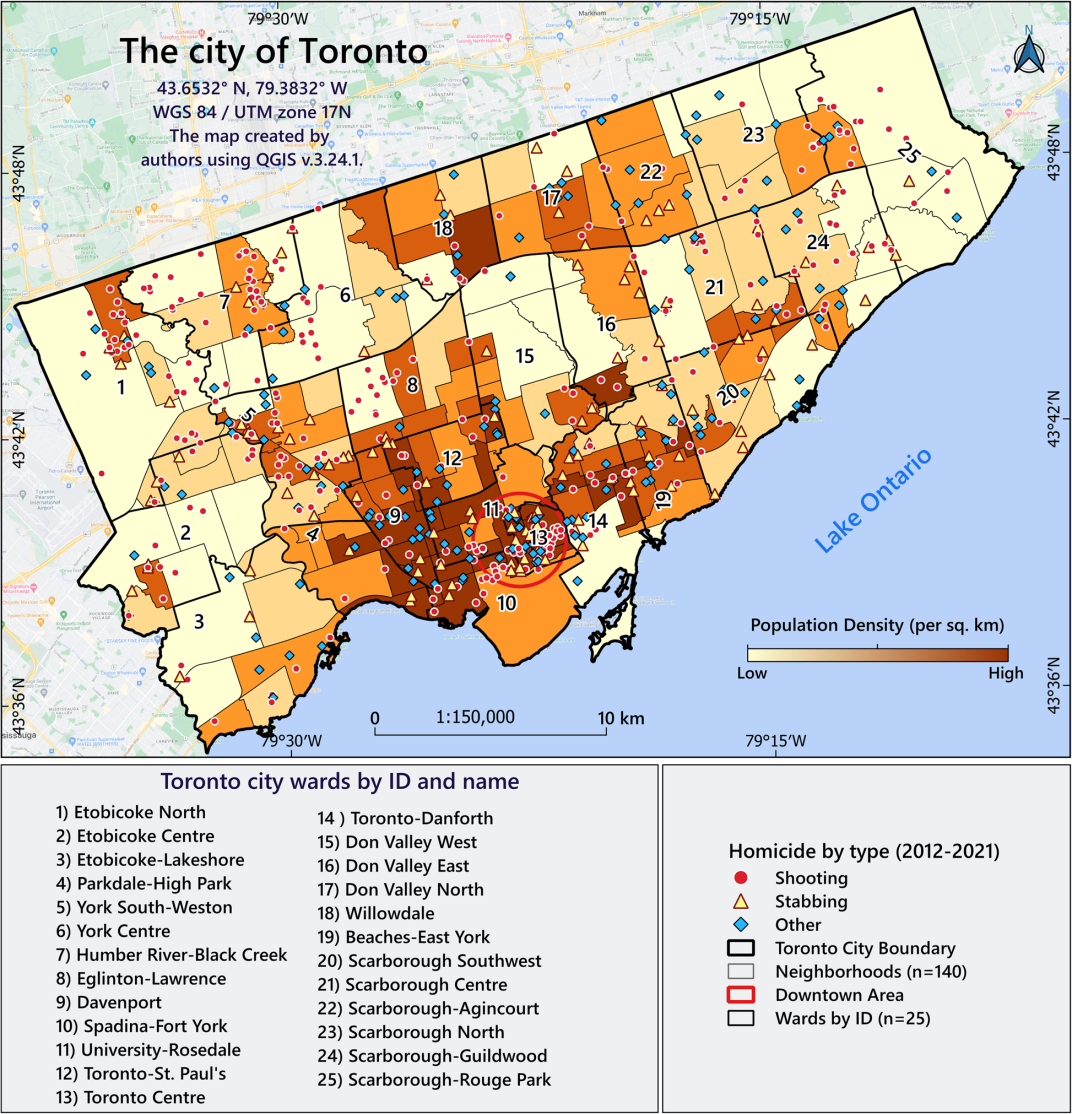
Geographic location of homicide incidents and population density in Toronto

Toronto, the capital of Ontario Province, is a major Canadian city along Lake Ontario’s north-western shore. The city covers an area of 630 km^2^ (243 mi^2^) and its population in 2020 was about 2,820,000 people with a density of 4,476 people per/km^2^ [76]. Toronto has 44 wards, 140 social planning neighbourhoods [77] and 29 police service divisions [78]. Figure 1 shows the homicide incidents by type between 2012-2021 in the city in relation to its spatial divisions. Further, the population density per km^2^ at the neighbourhood scale, i.e. the level at which our research was carried out.

### Datasets and selection of variables

**Fig. 2:**
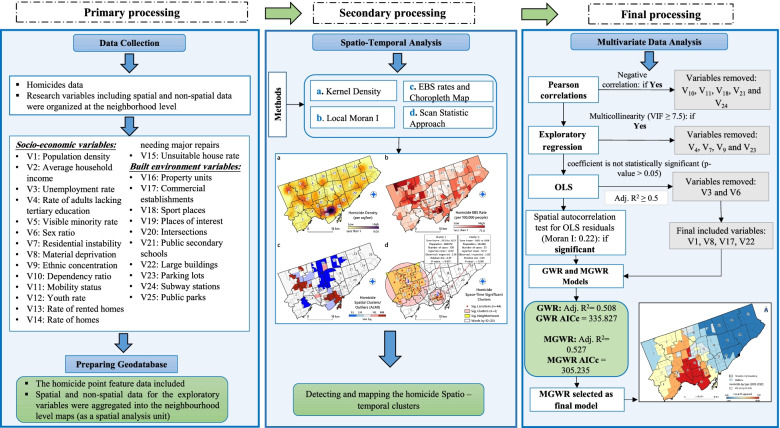
Methodological framework of this study

**Fig. 3:**
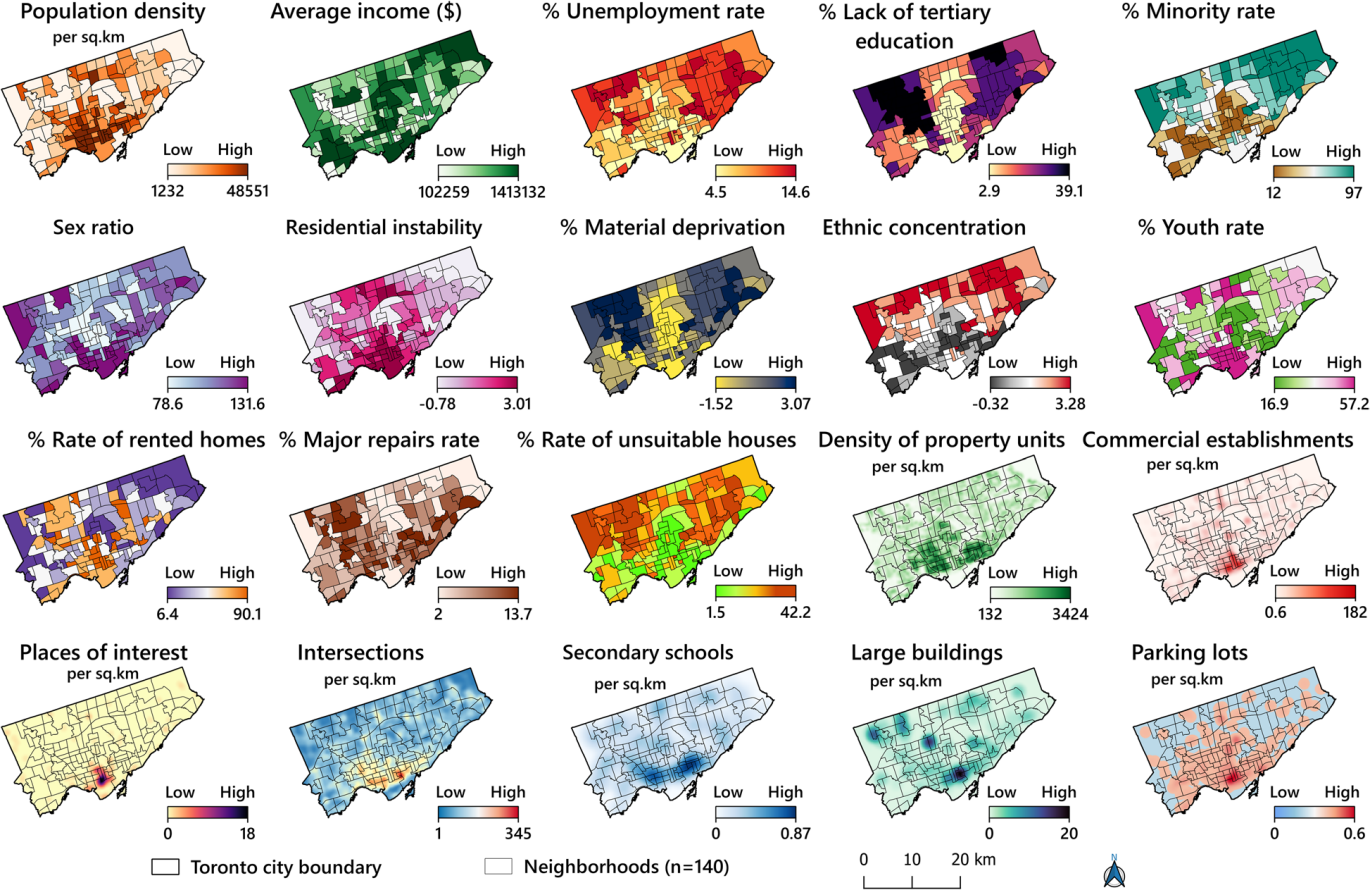
Spatial distribution of explanatory variables used for homicide modelling in the city of Toronto at the neighbourhood level

In a first step, a literature search identified 25 indicators related to socioeconomic characteristics and the built environment (Table 1). Pearson’s correlation was used to identify associated variables with homicide rates at the neighbourhood scale; five (dependency ratio, subway stations, sport places, public parks and mobility status) of the 25 variables did not significantly associate with the homicide rate and were removed from the rest of analyses (Supplementary File 1). Then, the exploratory regression analysis was conducted to remove the variables that had collinearity with each other, resulting in removing four variables (ethnic concentration, parking lots, rate of adults lacking tertiary education and residential instability) of the 20 remained variables with VIF bigger than 7.5 (Supplementary File 2). The exploratory regression model was run again with the remaining 16 variables as input, with the best model based on six variables (unemployment rate, population density, material deprivation, sex ratio, commercial establishments and large buildings) selected for the OLS regression (Supplementary File 3). Four variables (population density, material deprivation, commercial establishments and large buildings) remained for the geographical regression analysis (Supplementary File 4). Figure 2 shows the complete, methodological framework used in this study. Model implementation was thus carried out with only four independent variables, leaving three datasets to spatially analyse and explore the association between homicide rates and neighbourhood characteristics as follows:

1. The homicide dataset, containing 701 homicides recorded by Toronto Police Services (TPS) between 2012 and 2021, was extracted as geocoded points in a GIS shapefile [8]. It included the total number of homicides, killing locations, occurrence dates and type of homicide (shooting, stabbing etc.). These point data were aggregated to the neighbourhood polygon layer and used for analysis.
2. The socio-economic characteristics (Table 1) of 140 Toronto neighbourhoods were derived from the Toronto City government open data portal [79] and Ontario Marginalization Index (ON-Marg) (http://www.ontariohealthprofiles.ca). The population for the last Census of the study period (2016) was used to calculate the homicide rate. Since the socio-economic and built environment factors of neighbourhoods were presumed to become associated with crimes in the long term, the 2016 Toronto Census data and neighbourhood profiles were used as basis for selecting the independent variables [79, 80].
3. The built-environment indicators (Table 1) were extracted from the Toronto City government portal. Due to the importance of determining an accurate location of different places and built-environment features for spatial analysis, we calculated the spatial density per/km^2^ (based on the number of dwellings in each building) in each of these places (Table 1). This indicator allowed us to more accurately identify the areas of the city where large buildings are located. Figure 3 presents the spatial distribution and values (low to high) of each of the variables analysed in this study. It should be noted that the excluded variables by Pearson’s correlation have not been included in this figure.

**Table 1:** Built environmental and socio-economic factors used to explore association between homicide rate and neighbourhood characteristics in Toronto 2012-2021

**Dimension**	**Indicator**	**Description**	**Rationale**	**Status**	**Data** **source(s)**
***Socio-economic*** ***(N =*** ***15)***	V1: Population density	Dividing the total number of people by the total land area (km^2^)	Population density can be associated with high rates of violent crime in urban areas [40]	5	1
	V2: Average household income	Average after-tax income of households ($)	Low income and income poverty can play an important role in the occurrence of violent behaviour and crime [37]	3	1
	V3: Unemployment rate	Unemployed population/total population in the labour force aged 15 years and over ×100	There is an association between unemployment rates and the occurrence of violent behaviour, such as homicide [36]	4	1
	V4: Rate of adults lacking tertiary education	Population lacking tertiary education/total population aged 15 years and over ×100	Lack of tertiary education can associate with many crimes, including violent ones and homicide [31]	2	1
	V5: Visible minority rate	Total visible minority population/total population×100	Some studies [7, 39] have shown that violent crimes rates are higher among ethnic and racial minorities	3	1
	V6: Sex ratio	Total number of males/total number of females×100	Evolutionary behavioural models suggest that when the sex ratio is high (more available men than women), violence against women is more likely to occur [81]	4	1
	V7: Residential instability	This measure refers to area-level concentrations of people who experience high rates of family or housing instability, weighted average residential instability score - higher values mean more instability	Social disorganization theorists argue that residential instability can associate with the local violence crime rate by disrupting residential networks that are protective factors against crime [18]	2	2
	V8: Material deprivation	Material deprivation is closely connected to poverty and it refers to inability for individuals and communities to access and attain basic material needs. The indicators included in this dimension measure quality of housing, educational attainment and family structure characteristics [82]. Weighted average residential instability score – higher values mean more instability	Some studies have shown that homicide rates were higher in urban areas with higher material deprivation [83]	5	2
	V9: Ethnic concentration	Proportion of the population who self-identify as a visible minority, weighted average material deprivation score – higher values mean more deprivation	Some studies revealed that ethnic concentration exhibits a significantly positive but spatially different association with violent crime rates [30]	2	2
	V10: Dependency ratio	Dependency ratio (total population 0-14 and 65+ / total population 15 to 64), weighted average dependency score – higher values mean more dependency	Some studies have shown that in urban areas with high dependency rates, violent crime rates are also high [84, 85]	1	2
	V11: Mobility status	Mobility status 5 years ago – 25% sample data= total movers/total population × 100	High rates of geographic mobility (movement over time), High rates of geographical displacement in urban neighborhoods, while disrupting social organization, increase the possibility of crime [86, 87]	1	1
	V12: Youth rate	Youth 15-34 years old/total population×100	Some studies have shown that crime rates are higher than normal when the youth proportion in the population is high [23]; youth commit more crimes in easily accessible places and where there is less social control [7, 47, 48]	3	1
	V13; Rate of rented homes	Total number of renter households/total number of private households×100	The highest crime rates are in neighbour-hoods where a significant portion of all homes are rented [32]	3	1
	V14: Rate of homes needing major repairs	The number of private households whose dwellings are in need of major repairs/total number of private households×100	Urban decay and deterioration of buildings can turn neighbourhoods into areas where crime commonly occurs [62, 88]	3	1
	V15: Unsuitable house rate	Total number of private households who are living in unsuitable accommodations /Total number of private households×100	Poor housing condition is a potential risk factor for crimes and may be associated with areas with higher crime rates [89]	3	2
***Built-environment*** ***(N*** ***=*** ***10)***	V16: Property units	The total number of property units/total land area (km2)	As confirmed by some studies, the classic argument is that urban high density areas offers opportunities for violent crimes [90, 91]	3	3
	V17: Commercial establishments	The total number of commercial places/total land area (km^2^)	The rate of violent crimes, especially property theft, is higher in commercial spaces than in other spaces and may associate with homicide [41]	5	3
	V18: Sport places	The total number of sport places/total land area (km^2^)	Some studies [42, 47, 48] have shown that rates of violent crimes, especially those committed by young people, are high in public areas and sports places	1	2
	V19: Places of interest	The total number of places of interest/total land area (km^2^)	Recreational and interesting spaces may be a target for thieves due to overcrowding and disputes may lead to violence [61, 92].	3	3
	V20: Intersections	Dividing the total number of road intersections by total land area (km^2^)	Intersections provide opportunities for death by shooting, intentional car crashes or during escapes from crime scenes [44]	3	3
	V21: Public secondary schools	The total number of public secondary school locations/total land area (km^2^)	Schools are often examined in relation to delinquent behaviour [46, 93, 94] and this environment may protect youths at risk of delinquency [47]	3	3
	V22: Large buildings	The total number of buildings that includes >5 independent homes/total land area (km^2^)	Large, crowded buildings are more prone to all kinds of crime and violence [43, 95]	5	3
	V23: Parking lots	The total number of parking lots/total land area (km2)	Some studies have shown that the incidence of violent crimes, such as homicide, is higher in certain places such as parking lots [96]	2	3
	V24: Subway stations	The total number of subway stations/total land area (km2)	According to surveys, crime rates are high near subway stations [97]. High crowds at subway stations have the potential to lead to violent crimes such as robbery that lead to homicide [98]	1	3
	V25: Public parks	The total number of municipality public parks/total land area (km2)	Some studies have reported high rates of violence and violent crime in public parks [99, 100]	1	3

*WT* Wellbeing Toronto, *V* Variable

**Status in this study:** 1= Excluded by Pearson correlation; 2=Excluded by the first exploratory regression analysis, 3=Excluded by the second exploratory regression analysis, 4= Excluded by the OLS model, 5= Used in final model (GWR and MGWR)

**Data source(s):** 1=Wellbeing Toronto (http://toronto.ca/wellbeing); 2=Ontario Marginalization Index (ON-Marg), http://www.ontariohealthprofiles.ca/onmargON.php; 3=The City of Toronto’s Open Data Portal (https://www.open.toronto.ca)

### Data analysis

Kulldorff’s spatio-temporal analysis [101, 102] and spatial statistics [103] were used to map out the homicide patterns followed by application of OLS, GWR and MGWR to determine the associations between neighbourhoods characteristics and homicide rates. OLS regression was used to explore the associations globally [7, 104], with GWR and MGWR used for investigating the local changes of associations for each neighbourhood separately [30]. We also applied empirical Bayes smoothing (EBS) when mapping the homicide rate (Fig. 5-b) using the neighbourhood as spatial unit. The relevant population at risk typically varies across areas under investigation, which means that the precision of the raw homicide rate varies as well. This variance instability requires smoothing and we used the EBS technique to reduce the random fluctuations due to population size by computing the risk as a weighted sum of the raw rate for each unit and a prior mean. Thus, in this model, the underlying real rates were estimated by an assumed prior incident distribution based on the observed data [105–107]. The Jenks natural breaks classification [108] was used for generating a homicide rate map (Fig. 5b).

Purely temporal cluster analysis by SaTScan v.10 exclusively identifies time clusters in a particular time period and does not consider their geospatial patterns [109]. We first applied this approach using Poisson discrete scan statistic [110] to detect high-rates and low rate clusters with the length of time aggregation set at 1 year and the window size at 50%.

To visualize the degree of risk in the geographical areas under study, we used kernel density estimation (KDE), one of the non-parametric and distance-based techniques for calculation of the spatial intensity of point incidents [111–113]. Here, the value of each cell at the raster surface (image file format) refers to the number of values (incident density) [114, 115]. We used a 30-m cell size within a 3,500-m bandwidth displaying a smoothed spatial density map. The homicide density for each of the cells across the grid was estimated using equation 1 [116], while the spatial analyst mode in ArcGIS 10.8 (ESRI. Redlands, CA, USA) was used to conduct the KDE of spatial density of the homicides (Fig. 5-a). The KDE calculations are expressed by equation 1.

1 $$f\left(x, y\right)=\frac{1}{{nh}^{2}}\sum_{i=1}^{n}K\left(\genfrac{}{}{0pt}{}{{d}_{i}}{h}\right)$$

where; $f\left(x, y\right)$ is is the density estimate at the location $\left(x, y\right)$; $n$ the number of observations (homicides in this case); $h$ the bandwidth or the kernel size; $K$, is the kernel function; and ${d}_{i}$ the distance between the location $\left(x, y\right)$ and the location of the $i$ ^th^ observation.

Waldo Tobler's First Law of Geography states that "Everything is related to everything else, but near things are more related than distant things " [117], which encapsulates the concept of spatial dependence that can be estimated by autocorrelation techniques. The global autocorrelation techniques can identify any non-random distribution of clusters but do not tell where they are situated, which is revealed by local autocorrelation [110]. We used Global Moran's Index (GMI) [40] and Anselin's Local Moran's Index (ALMI) [41] since they are generally more accurate concerning measuring autocorrelation than other statistics [34, 37, 40, 41]. We used GMI to explore the general, spatial pattern of homicide rates in Toronto and also to test the residual values of the OLS results. To discover spatial autocorrelation, the spatial weights matrix [115] was used to conceptualize the spatial relationships, which is an essential element in the construction of spatial autocorrelation statistics in GIS [115]. The calculation steps of the ALMI and GMI models were done by equations 2 and 3.

GMI, an index of spatial autocorrelation is mathematically expressed as follows:

2 $$I = \frac{{N{\Sigma }_{ij} W_{ij} \left( {X_{i} - \overline{X}} \right)\left( {X_{j} - \overline{X}} \right)}}{{{\Sigma }_{ij} W_{ij} {\Sigma }_{i} \left( {X_{i} - \overline{X}} \right)^{2} }}$$

where N is the number of neighbourhoods, *Xi* thehomicide rate at area *I*; $\overline{X }$ the mean value of the homicide in the study neighbourhood; and ${W}_{ij}$ elements of a spatial lag operator *W* (spatial weights of matrix *W*).

ALMI an index of local spatial autocorrelation, is mathematically expressed as follows:

3 $$I_{i} = \frac{{\sum\nolimits_{j = 1}^{n} {w_{ij} \left( {x_{i} - \overline{x}} \right)\left( {x_{j} - \overline{x}} \right)} }}{{\frac{1}{n}\sum\nolimits_{i = 1}^{n} {\left( {x_{i} - \overline{x}} \right)}^{2} }},i \ne j$$

where n is the number of neighbourhoods; *x*_*i*_ and *x*_*j*_ the homicide rate in neighbourhood *i* and *j*, respectively; $\overline{x }$ the average of the reported homicide rate in all neighbourhoods; and *w*_*ij*_ the spatial weight matrix corresponding to neighbourhoods *i* and *j*; and *I* the local Moran’s *I* [103, 118, 119].

Spatio-temporal scan statistics were used to identify potential clustering of homicides in both space and time. This type of statistics, introduced by Naus in 1965 [120] and further developed by Kulldorff by 1997 [121], has since been applied in various types of crime analysis studies [122]. This approach can detect spatial clusters irrespective of any predefined geographical boundaries by combining any number of close locations into the same cluster in predefined periods [109]. It was designed to test whether or not an event is randomly distributed over space and time with the ability to repeat similar analyses [123]. Relative risk (RR), Log-likelihood ratio (LLR) and the Monte Carlo test, described in detail in previous studies [110], support the interpretation of space-time analysis in scan statistics. The Poisson probability model [102], which is a discrete scan statistic, was used to analyse temporal and spatio-temporal clustering in areas with high rates of total homicide incidents. The maximum window size of spatial and temporal analysis was adjusted to 50% of the population at risk in the study area during the period of study. The null hypothesis of no clusters was rejected at the simulated value of *p* ≤0.05 for the primary clusters [124]. QGIS v.3.24.1 was used to visualize the outputs of scan statistics.

### Linear and geographically weighted regression

An OLS multivariate regression model was employed to explore the global relationship between the homicide EBS rates (dependent variable) and the independent variables (Table 1). Before implementing the OLS model, Pearson’s correlation [125] and exploratory regression [115] were used to identify the global variables and to determine any multi-collinearity among independent variables. The MGWR model was used to improve our understanding of the spatially varying relationships between the homicide EBS rate and the explanatory variables included in the OLS model. Unlike traditional, global regression modelling techniques, which assume that the relationships examined through the model’s parameters are constant, MGWR allows variation across space [126]. Additionally, in contrast to GWR, which assumes that the local relationships within each model vary at the same spatial scale, MGWR allows the conditional relationships between the response variable and the different predictor variables to vary at different spatial scales, i.e. the bandwidths that indicate the range over which data are borrowed can vary by parameter surface [126]. The calculation steps of the GWR and MGWR models were done by equations 4 and 5. For a GWR model, the linear regression model is as follows:

Assuming that there are n observations, for observation

4 $$i \in \left\{ {1,2,...,n} \right\}\, {\text{at location}}\,(u_{i} ,v_{i} ),y_{i} = \beta_{0} \left( {u_{i} ,v_{i} } \right) + \mathop \sum \limits_{j = 1}^{m} \,\beta_{j} (u_{i} ,v_{i} )x_{ij} + \varepsilon_{i}$$

where $\beta_{0} \left( {u_{i} ,v_{i} } \right)$ is the intercept; *X*_*ij*_ the *j*^th^ predictor (independent) variable: $\beta_{j} (u_{i} ,v_{i} )$ the *j*^th^ coefficient; $\varepsilon_{i}$ the error term; and *y*^*i*^ the response variable (Crime EBS rate).

For a MGWR model, the linear regression model is as follows:

Assuming that there are n observations, for observation

5 $$i \in \left\{ {1,2,...,n} \right\}\, {\text{at location}}\,(u_{i} ,v_{i} ),y_{i} = \beta_{0} \left( {u_{i} ,v_{i} } \right) + \mathop \sum \limits_{j = 1}^{m} \,\beta_{bwj} (u_{i} ,v_{i} )x_{ij} + \varepsilon_{i}$$

where *bwj* in $\beta_{bwj}$ indicates the bandwidth used for calibration of the *j*^th^ conditional relationship.

Gaussian model was used to run the GWR and MGWR models [126] with the introduction of locations (identified by ID-labels), coordinates variables (x and y), four independent variables (Supplementary file 4) and the EBS homicide rate as the dependent variable. To select an optimal bandwidths in both models for comparison purposes, the adaptive Bisquare spatial kernel method [126] was used and the Golden Section mode [126] applied as a weighting scheme for calibrating both models. The corrected Akaike Information Criterion (AICc) was used as an optimization criterion in the calibration of the GWR and MGWR models, and local variation inflation factors (VIF) [127] were applied to evaluate multi-collinearity amongst explanatory variables. It was also possible to test the statistical significance of each surface of parameter estimates produced by GWR and MGWR via random sampling methods. In this study, a Monte Carlo test with 1,000 iterations [126]was applied to evaluate the spatial variability of each surface of parameter estimates produced by the MGWR model. A pseudo *p*-value <0.05 indicated that the observed spatial variability of a coefficient surface was significant at the 95% CL (i.e. non-random).

## Results

### Temporal clusters

**Fig. 4:**
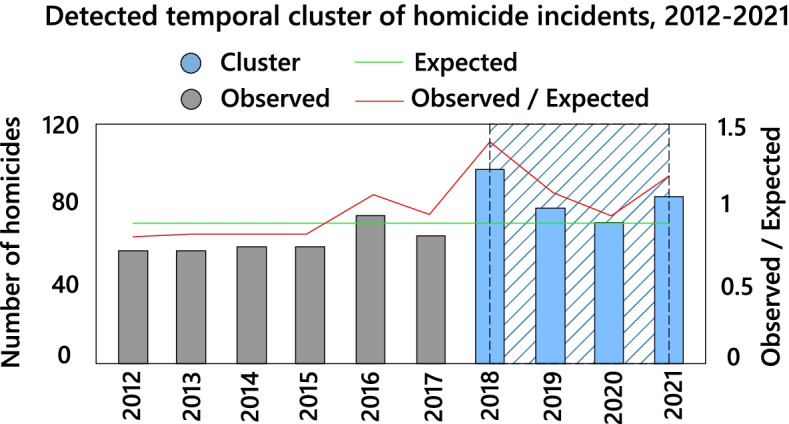
Temporal clusters of homicide incidents in the city of Toronto, 2012-2021

There were 701 homicides in Toronto in the 2012-2021 period. The lowest number (57 cases) occurred in 2012 and the highest (98 cases) in 2018. Although the number of homicides decreased from 2018 to 2021, it had increased 32.14% by 2021 compared to 2012. An average of 70 homicides per year occurred during the study period. The results of the purely temporal analysis indicated that high-rate clusters of total homicides were predominantly distributed in the period 2018-2021 (Fig. 4). In the study period, the average age of the victims was 33 years and 75.7% of them were men. Death by shooting (52.35%) was the most common type of homicide in the study period.

### Spatial and spatio-temporal clusters

**Fig. 5:**
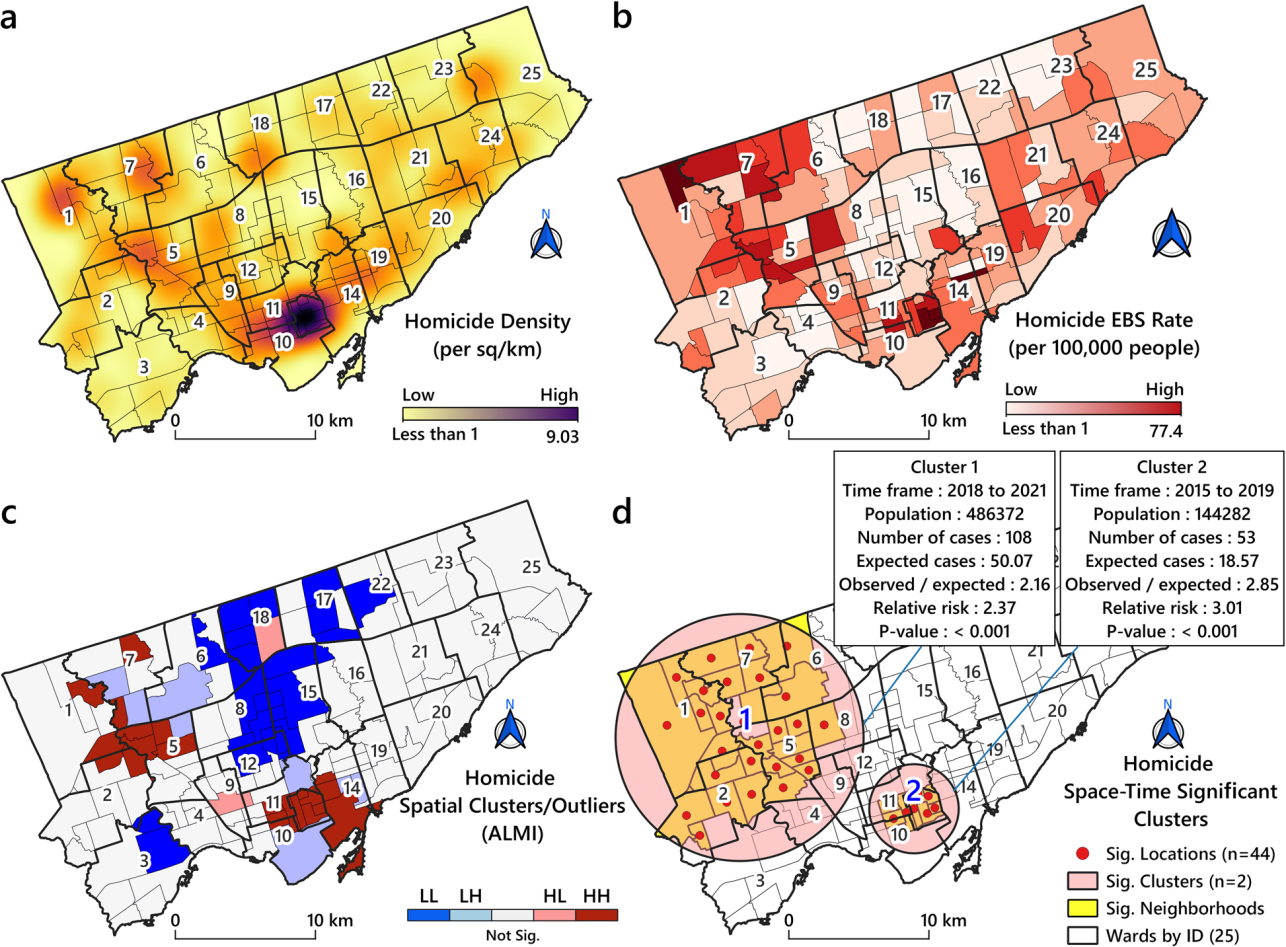
Distribution of homicide by neighbourhood in Toronto2012-2021. **A** Homicide density per km2; **B** Homicide EBS rates; **C** Homicide spatial patterns (Low-Low (LL), Low-High (LH), High-Low (HL) and HH; **D** Two homicide spatio-temporal clusters were identified in this study

Figure 5-A shows the density and location of the homicides for the 2012-2021 period expressing the former as number per km^2^. According to this map, downtown Toronto had the highest number of homicides per km^2^ (9.03). Areas in the North (Humber River and Black Creek, ID=7) and Northwest (Etobicoke North, ID=1) also showed high homicide rates (Fig. 5-B). However, this particular map only deals with population density and does not take into account the issue of neighbourhoods and the proximities of different geographical units. However, based on the following results (Moran's *I* = 0.22, Z-score = 5.8, *p* = 0.00), GMI revealed that the global spatial pattern of homicides rate in Toronto during the study period was autocorrelated and clustered. Figure 5-C maps the homicide clusters and outliers using EBS rates and the ALMI method. According to this map, downtown Toronto and the area Etobicoke North (ID=1) had two High-High (HH) clusters that were spatially autocorrelated. We identified two spatio-temporal clusters: the first cluster (RR = 2.37, OE = 2.16 and *p*<0.05) formed in Etobicoke North (ID=1) during the years 2018-2021. The second (RR = 3.01, Observed/Expected (OE) = 2.85 and *p* <0.05) covered the city centre during the years 2015-2019. The spatio-temporal homicide patterns are shown in Figure 5-D.

### Pearson’s correlation, ER and OLS model

As reported in Supplementary File 3, the VIF values of all dependent variables derived from the second exploratory regression analysis were <7.5, indicating that there was no multicollinearity. Neighbourhoods (e.g., Sunnylea) where no homicides occurred during the study period were identified as outliers and excluded from the analysis by OLS, GWR, and MGWR. The results of Pearson’s correlation test showed that there was a global, significant relationship between the homicide rate and the selected variables, e.g., between homicide rate and the spatial density of large buildings (correlation = 0.56, *p*<0.01). However, Pearson’s test does not show correlation between variables in their geographical context, which can vary in terms of strength and direction in different neighbourhoods. The OLS method, on the other hand, shows the associations between homicide rate, intercept and independent variables in their geographical context (Supplementary file 4). Our findings based on OLS calculations show that the population density, the material deprivation index, the commercial establishments and the density of large buildings were significantly associated with high homicide rates (Supplementary File 4).

Naturally, the strength of this association varied in different areas and some variables were more strongly associated with high homicide rate. R^2^ and the adjusted R^2^ (Adj.R^2^) obtained from the OLS model explained 53% and 50%, respectively, of the total variance of homicide rates within the neighbourhoods. Moran's *I* statistic showed a positive, significant autocorrelation for the residuals values of the OLS model results (*I* = 0.14, z-score = 2.18, *p*<0.05) which rejects the random distribution of residual values. However, the non-random pattern of the residuals impairs their independence in the OLS model. To address this limitation, GWR and MGWR methods were applied.

### GWR model results

The descriptive results of the GWR for homicides are provided in Tables 2 and 3. Adj R^2^ of the GWR was 0.51, signifying a 0.6% higher value than that obtained by the OLS model and the GWR also produced a decreased AICc (309.53). Thus, compared to the OLS model, the GWR increased the explanatory level to 54% and 51%, respectively, of the variations in the observed homicide rates across different neighbourhoods.

**Table 2:** Summary statistics of GWR model estimated coefficients of local terms for homicides

**Variable**	**Bandwidth**	**Mean**	**STD**	**Minimum**	**Median**	**Maximum**
Intercept	123	-0.027	0.060	-0.146	-0.046	0.075
Population density	123	-0.267	0.011	-0.300	-0.265	-0.245
Material deprivation	123	0.424	0.083	0.311	0.437	0.555
Commercial establishments	123	0.350	0.062	0.251	0.333	0.526
Large buildings	123	0.401	0.042	0.288	0.404	0.480

**Table 3:** Model specifications and diagnostics indicators for the fitted GWR model

**Diagnostic name**	**Value**		**Value**
Residual sum of squares	64.627	AICc	309.528
Effective number of parameters (trace (S))	8.459	BIC	335.827
Degree of freedom (n – trace (S))	131.541	**R**^**2**^	**0.538**
Sigma estimate	0.701	**Adj. R**^**2**^	**0.508**
Log-likelihood	-144.541	Adj. alpha (95%)	0.030
Degree of Dependency (DoD)	0.894	Adj. critical t value (95%)	2.199
AIC	308.000		-

### MGWR model results

The descriptive results of the MGWR for homicides are provided in Tables 4 and 5. Table 6 compares the diagnostics indicators of all three methods used. AdjR^2^ of the MGWR was 0.53, representing a 4.35 and 3.74% increase, respectively, from that in the OLS and GWR models, (Table 6). The MGWR also produced a better AICc (305.24) indicating that the MGWR is even more suitable as it explains 56 and 53%, respectively, of the variations in observed homicide rate. Moran's *I* statistic was negative and had no significant autocorrelation for the MGWR residuals (*I* = -0.021, z-score = -0.36, *p*>0.05), which is a random pattern that confirms their independence.

**Table 4:** Summary statistics of MGWR model estimated coefficients of local terms for homicides

**Variable**	**Bandwidth**	**Mean**	**STD**	**Min**	**Median**	**Max**	**Monte Carlo test**
Intercept	139	0.006	0.012	-0.030	0.007	0.030	0.840
Population density	139	-0.254	0.005	-0.272	-0.252	-0.249	0.905
Material deprivation	123	0.415	0.079	0.301	0.427	0.522	0.143
Commercial establishments	70	0.375	0.247	0.068	0.301	0.036	0.002
Large buildings	139	0.430	0.009	0.409	0.432	0.447	0.905

**Table 5:** Model specifications and diagnostics indicators for the fitted MGWR model

**Diagnostic name**	**Value**		**Value**
Residual sum of squares	61.667	AICc	305.235
Effective number of parameters (trace (S))	9.432	BIC	334.066
Degree of freedom (n – trace (S))	130.568	**R**^**2**^	**0.560**
Sigma estimate	0.687	**Adj. R**^**2**^	**0.527**
Log-likelihood	-141.259	-	-
Degree of Dependency (DoD)	0.872	-	-
AIC	303.380	-	-

**Table 6:** Model comparison

**Model**	**AIC**	**AICc**	**R**^**2**^	**Adj. R**^**2**^	**Increased Adj. R**^**2**^**(%)**
**OLS**	1027.85	1028.95	0.526	***0.505***	-
**GWR**	309.528	335.827	0.538	***0.508***	0.003= 0.6%
**MGWR**	303.380	305.235	0.560	***0.527***	0.022=4.35%, 0.019=3.74%

**Fig. 6:**
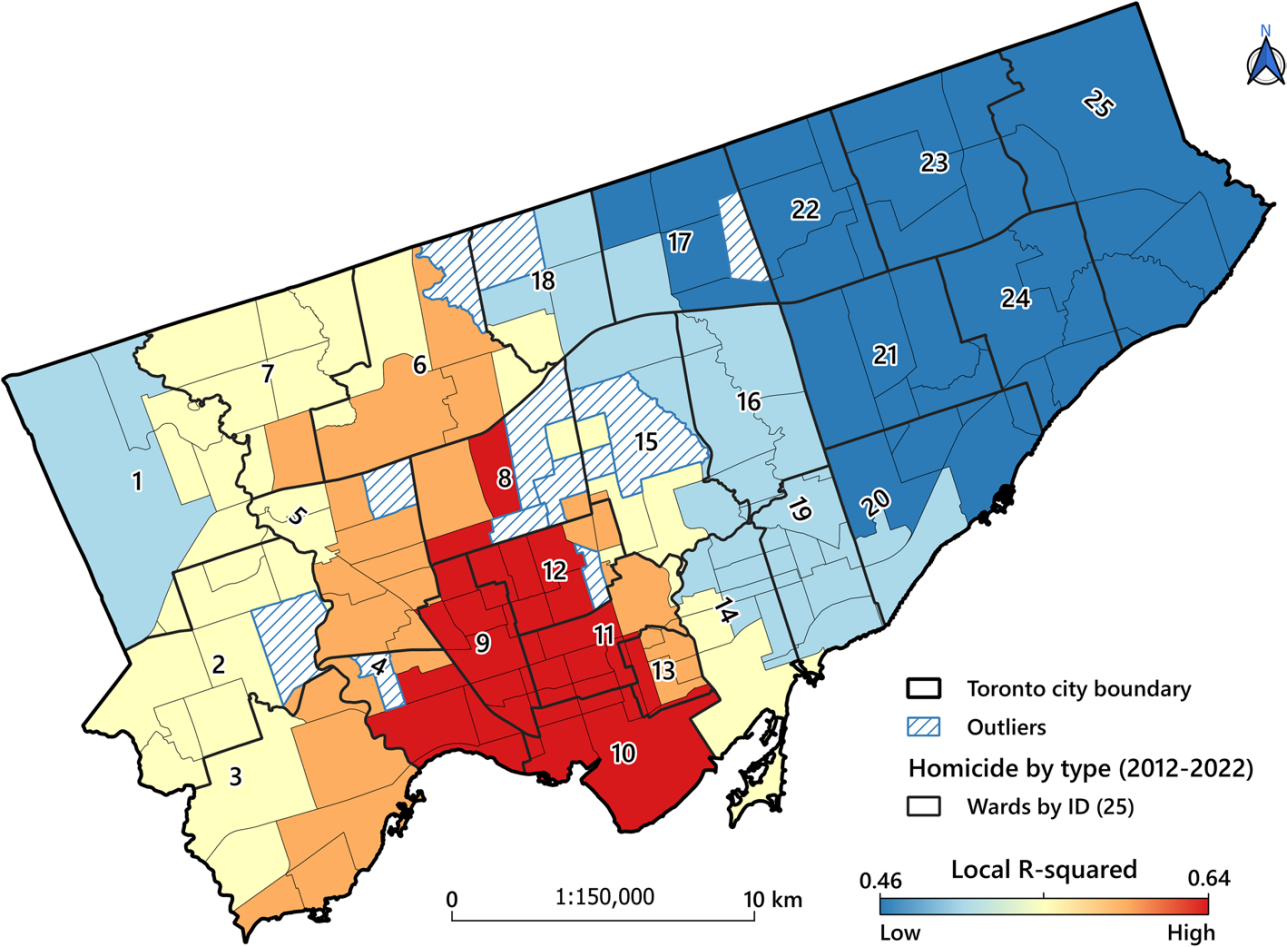
Spatial distribution map of adjusted local R^2^ of the MGWR model

The spatial results of GWR and OLS models are not visualized in this article; however, they are presented in Tables 2 and 3 and Supplementary file 4. Geographical mapping of the estimated locally weighted R^2^contributes to the understanding of how well the MGWR model fits observed homicide rate in the different neighbourhoods. Figure 6 depicts the distribution of local R^2^, which is heterogeneously distributed. In general, MGWR operates well in the downtown area, with R^2^ values over 0.64. Indeed, Eglinton-Lawrence (ID=8), Davenport (ID=9), Spadina-Fort York (ID=10), University Rosedale (ID=11), Toronto St. Paul’s (ID=12) and small part of the Toronto Centre (ID=13) wards included neighbourhoods associated with R^2^ values over 0.64. Neighbourhoods in the western and eastern ends of the city were found to be associated with lower local R^2^ values. Some of the neighbourhoods in Scarborough (IDs= 17 and 20-25) and Etobicoke North (ID=1) showed particularly low R^2^ (0.46), which suggests that additional explanatory factors might be associated with the homicide rate in these neighbourhoods.

**Fig. 7:**
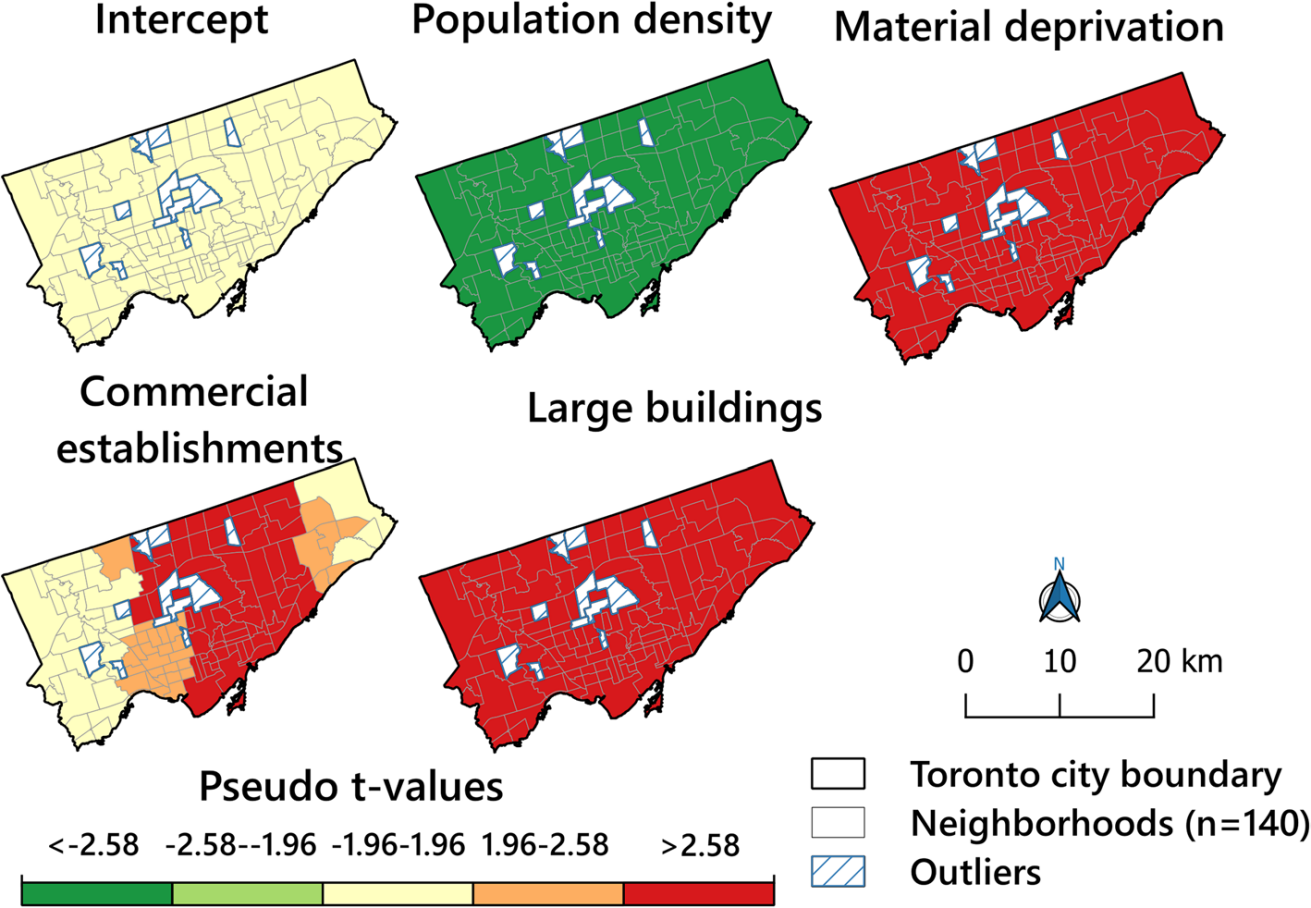
Pseudo t-values for intercept and independent variables

**Fig. 8:**
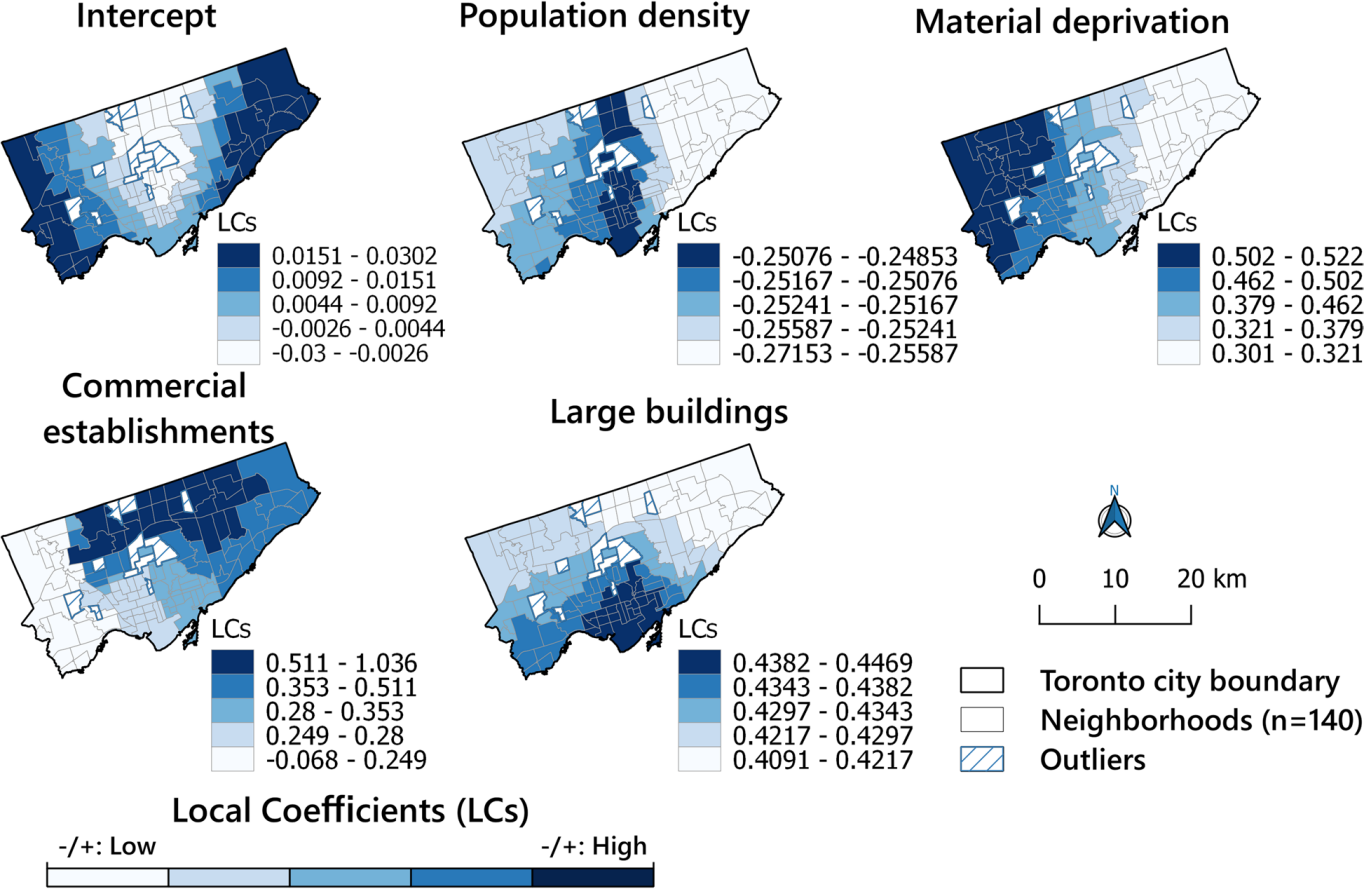
MGWR local coefficients for intercept and independent variables

In the MGWR model, the significance of locally varying coefficients for the independent variables can be visualised through pseudo *t-*statistics [30]. Figure 7 shows the spatial distribution map of pseudo *t*-values for the intercept and each independent variable in the Toronto City. In figure 7, the non-significant relationships are shown in light yellow; significant positive relationships in orange/red; and significant negative relationships in light green/green. Figure 8 visualises local coefficients for the variables identified significant in Figure 7. It essentially reveals how the direction and strength of the association between the dependent and each independent variable varied over the total surface. Examining both pseudo *t*-values for the surface in Figure 7 and coefficient maps in Figure 8 yielded useful insights into the spatial variation of associations. In fact, the map shows that material deprivation and large buildings are positively associated with the homicide rate; however, population density is negatively associated. Finally, commercial establishments followed different directions regarding the association with the homicide rate in different neighbourhoods.

## Discussion

The study aimed to explore the spatial patterns of homicide rate at the neighbourhood level in Toronto, the largest urban area of Canada. Our findings show that the homicide rate increased during the study period (2012-2021) and reached a high in 2018. Importantly, uncommon events may have distorted the study results, particularly due to the 2018 attack in the North York neighbourhood and City Centre of Toronto, where pedestrians were deliberately struck by a van resulting in 10 deaths [128], but also the fact that eight homicides discovered during the seven-year period from 2010 to 2017 were finally found to have been committed by a serial killer [129]. However, even after subtraction of these particular cases, the average homicide rate in Toronto climbed over the last decade.

Felson and Clark [130] and Brantingham [26] point out that consistent high crime rates tend to attract more crime leading to the “*law of crime concentrations at places*”, something that has been verified in Toronto [131]. Hirschfield and Bowers [68] confirm that homicides are non-random occurrences in urban areas and indeed repeated in areas with special characteristics, something which is supported by our study as well as the majority of investigations [27, 30, 48, 132]. Although violence rates in different periods and different parts of a city can be quite different [133], many scientists [29] confirm that city centres offer opportunities for crime due to their diverse social and economic attractions, while Charron [47] also notes that commercial areas bring together large numbers of people whose interactions can be associated with violent crimes. Our ALMI maps support these findings, as they reveal statistically significant spatial homicide clusters in many various parts of Toronto where spatial HH clusters of homicide rates formed during the study period, results which also are in line with previous research in Toronto by Wang et al. [30] and Charron [48].

The GWR results (R^2^= 0.54) confirm the association between some socio-economic variables and the built environment and, as MGWR allows variability at different spatial scales, conditional relationships between the response variable and the different predictor variables could be traced (e.g., the local R^2^ surface revealed the extent at which the regression model fits observed homicide rate in different neighbourhoods). However, in this respect, our findings differed from those by Wang et al. [30]. In their study, the R^2^ values were particularly high in north-eastern Toronto, while we reached the highest values in the central parts and downtown area in our study (Fig. 6). However, this does not mean that the results are inconsistent as spatial heterogeneity with regard to homicides is not uncommon as shown by Graif and Sampson [56]. Pseudo *t* values and local coefficients also show that some variables, such as population density, material deprivation, commercial establishments and large buildings (including >5 households) density were all associated with high homicide rates in some neighbourhoods. Also, the strength and direction of local coefficients varied in different neighbourhoods, e.g., while the presence of large buildings, as commonly in city centres, were positively associated with high homicide rates. In addition, material deprivation can be associated with the high rates as they were in the city's north-western neighbourhoods. Our findings based on the MGWR model revealed that there was a strong local correlation between a high homicide EBS rate, population density and density of large buildings in most parts of the city such as central neighbourhoods. According to Colquhoun [95], areas with high population density and a concentration of large buildings can be associated with increased violent crime and homicides. Newman [134] emphatically states that when building density increases with more households in the same building, the sense of belonging decreases and crime opportunity increases. In his opinion, this occurs when buildings are poorly designed and characterised by low-income households, and environmental improvements can be an effective way to prevent crime in densely populated areas [134]. However, in our study, only some associations were identified.

Our findings based on the MGWR model also revealed that there were an association between the homicide rate and material deprivation in most parts of the city, particularly in areas with high unemployment rates, low levels of tertiary education and high rates of dilapidated, unsuitable housings (Fig. 3). This conclusion is echoed by a large number of authors [31, 58, 62, 63, 95, 135, 136], who also note that the number of various crimes grows with increased deterioration, i.e. poor areas with dwellings in need of major repair (burnout and destruction of the physical environment) populated by people with low income and a low rate of tertiary education areas. The results by Lockwood [92] and those reported by Ingram et al. [58], also confirm a significant association between homicide and both poverty in urban areas. According to Lockwood, poverty and poor areas are associated with more violent crimes. Kitchen and Schneider [133] agree regarding the role of socioeconomic disadvantage for violent crime rates in specific neighbourhoods, as do Tita et al. [137] and others [138].

Finally, the results of pseudo t-values we obtained from the MGWR model showed that the associations between homicide and areas with a high density of commercial establishments in most parts of Toronto (Figs. 7 and 8). As previous studies have confirmed [30, 41–45, 47, 48], the parts of a city characterized by a high density of commercial establishments, are attractive centres for all types of crimes that can be associated with violent crimes.

### Limitations and future research areas

While the study has contributed to a better understanding of the socio-economic and built environment factors associated with homicides in Toronto, there are some limitations that need to be acknowledged. First, only data reported by Toronto Police Service were analysed and some homicides may not have been reported to the police for various reasons (such as fear, dissatisfaction with the police, etc.) [139]. Neither did we have access to data for areas outside of the City of Toronto [30] nor were detailed data for any offenders and victims available. Knowledge of the place of residence of killers and victims could deepen spatial analysis and provide a better understanding of homicide spatial variations. It is also possible that factors outside the artificial boundaries of neighbourhoods could be associated with high homicide rates. Cross-border variables could play a role and need to be investigated. Second, in this study, we only used aspects of the spatial distribution of the homicides, while data on uncertain geographic contexts and spatial behaviour of offenders were not considered (e.g., the killers’ move from home to the crime scene). Future studies might be able to use interviews to get more detailed data about the spatial behaviour of offenders, thereby assisting spatio-temporal analyses. Third, we used the population data of 2016 as the middle point of the study period. However, this cannot be a serious limitation of the associations found in this study because the data of 2016 for calculating the independent variables were also used. On the other hand, it can underestimate the homicide rate of neighborhoods which grew at a faster pace between 2012 and 2021. Fourth, the current research has manifested some urban indicators associated with a high homicide rate, but this kind of research cannot show any causality inference and many of the associations we estimate could be a product of inverse causality. Future research with different study designs is needed to find the factors influencing the homicide rate in different urban neighbourhoods. Finally, choosing the neighbourhood level as the basic unit of analysis may cause the modified areal unit problem.

## Conclusions

By applying geographical regression methods to identify socioeconomic and built environment factors associated with homicide, we expect the current study to improve the understanding of which factors are associated with the occurrence and recurrence of crime in each neighbourhood. Urban planners need to address the problems in downtown and north-western areas of Toronto, in particular with respect to dense urban areas with high proportion of large urban buildings, areas with high deprivation rates and urban areas characterized by a concentration of commercial establishments. Reducing violent crime requires long-term integrated strategies (socioeconomic and built-environment).

## Supplementary Information

**Additional file 1.**

**Additional file 2.**

**Additional file 3.**

**Additional file 4.**

## Data Availability

The datasets analysed during the current study come from the public databases of Wellbeing Toronto [http://toronto.ca/wellbeing], Open Toronto [https://www.open.toronto.ca] and the open data dashboard of Toronto Police [https://data.torontopolice.on.ca/pages/homicide].

## Abbreviations

GIS: Geographic Information Systems
TPS: Toronto Police Service
VIF: Local Variation Inflation Factors
OLS: Ordinary Least Square
GWR: Geographically Weighted Regression
MGWR: Multi-scale Geographically Weighted Regression
KDE: Kernel Density estimation
LCs: Local Coefficients
AIC: Akaike’s Information Criterion
GMI: Global Moran’s Index
ALMI: Anselin’s Local Moran's Index
AICc: Corrected Akaike Information Criterion
EBS: Empirical Bayes Smoothing
