# Supplementary material for: Homicide rates are spatially associated with built environment and socio-economic factors: a study in the neighbourhoods of Toronto, Canada
Source: BMC Public Health. 2022 Aug 4;22:1482. doi: 10.1186/s12889-022-13807-4 (PMC9351166; doi:10.1186/s12889-022-13807-4)
Supplement: Supplementary file 4 — Additional file 4. [file 12889_2022_13807_MOESM4_ESM.pdf]

Summary of OLS Results - Model Variables

Variable	Coefficient [a]	StdError	t-Statistic	Probability [b]	Robust_SE	Robust_t	Robust_Pr [b]	VIF [c]
Intercept	6.900171	13.434027	0.513634	0.608367	11.715873	0.588959	0.556890	--------
DEPRIVATIO	8.414183	1.494425	5.630382	0.000000*	1.209282	6.958001	0.000000*	2.927707
COMMERCIAL	0.154051	0.053253	2.892806	0.004465*	0.049563	3.108197	0.002309*	2.944452
HBINGS	1.554564	0.417874	3.720175	0.000300*	0.449052	3.461878	0.000731*	2.593071
UNEMPRATIO	-1.193093	0.679036	-1.757040	0.081218	0.653717	-1.825093	0.070234	2.716557
SEXRATIO	0.264589	0.135139	1.957902	0.052333	0.121105	2.184800	0.030648*	1.551234
POPDENSITY	-0.000624	0.000202	-3.087945	0.002460*	0.000165	-3.789588	0.000235*	2.059071

OLS Diagnostics

Input Features:	Homicide	Dependent Variable:	H_EBS_RATE
Number of Observations:	140	Akaike's Information Criterion (AICc) [d]:	1028.957784
Multiple R-Squared [d]:	0.526423	Adjusted R-Squared [d]:	0.505059
Joint F-Statistic [e]:	24.640259	Prob(>F), (6,133) degrees of freedom:	0.000000*
Joint Wald Statistic [e]:	267.734444	Prob(>chi-squared), (6) degrees of freedom:	0.000000*
Koenker (BP) Statistic [f]:	6.066248	Prob(>chi-squared), (6) degrees of freedom:	0.415810
Jarque-Bera Statistic [g]:	23.727580	Prob(>chi-squared), (2) degrees of freedom:	0.000007*

Notes on Interpretation

* An asterisk next to a number indicates a statistically significant p-value (p < 0.01).

[a] Coefficient: Represents the strength and type of relationship between each explanatory variable and the dependent variable.

[b] Probability and Robust Probability (Robust_Pr): Asterisk (*) indicates a coefficient is statistically significant (p < 0.01); if the Koenker (BP) Statistic [f] is statistically significant, use the Robust Probability column (Robust_Pr) to determine coefficient significance.

[c] Variance Inflation Factor (VIF): Large Variance Inflation Factor (VIF) values (> 7.5) indicate redundancy among explanatory variables.

[d] R-Squared and Akaike's Information Criterion (AICc): Measures of model fit/performance.

[e] Joint F and Wald Statistics: Asterisk (*) indicates overall model significance (p < 0.01); if the Koenker (BP) Statistic [f] is statistically significant, use the Wald Statistic to determine overall model significance.

[f] Koenker (BP) Statistic: When this test is statistically significant (p < 0.01), the relationships modeled are not consistent (either due to non-stationarity or heteroskedasticity). You should rely on the Robust Probabilities (Robust_Pr) to determine coefficient significance and on the Wald Statistic to determine overall model significance.

[g] Jarque-Bera Statistic: When this test is statistically significant (p < 0.01) model predictions are biased (the residuals are not normally distributed).

Variable Distributions and Relationships

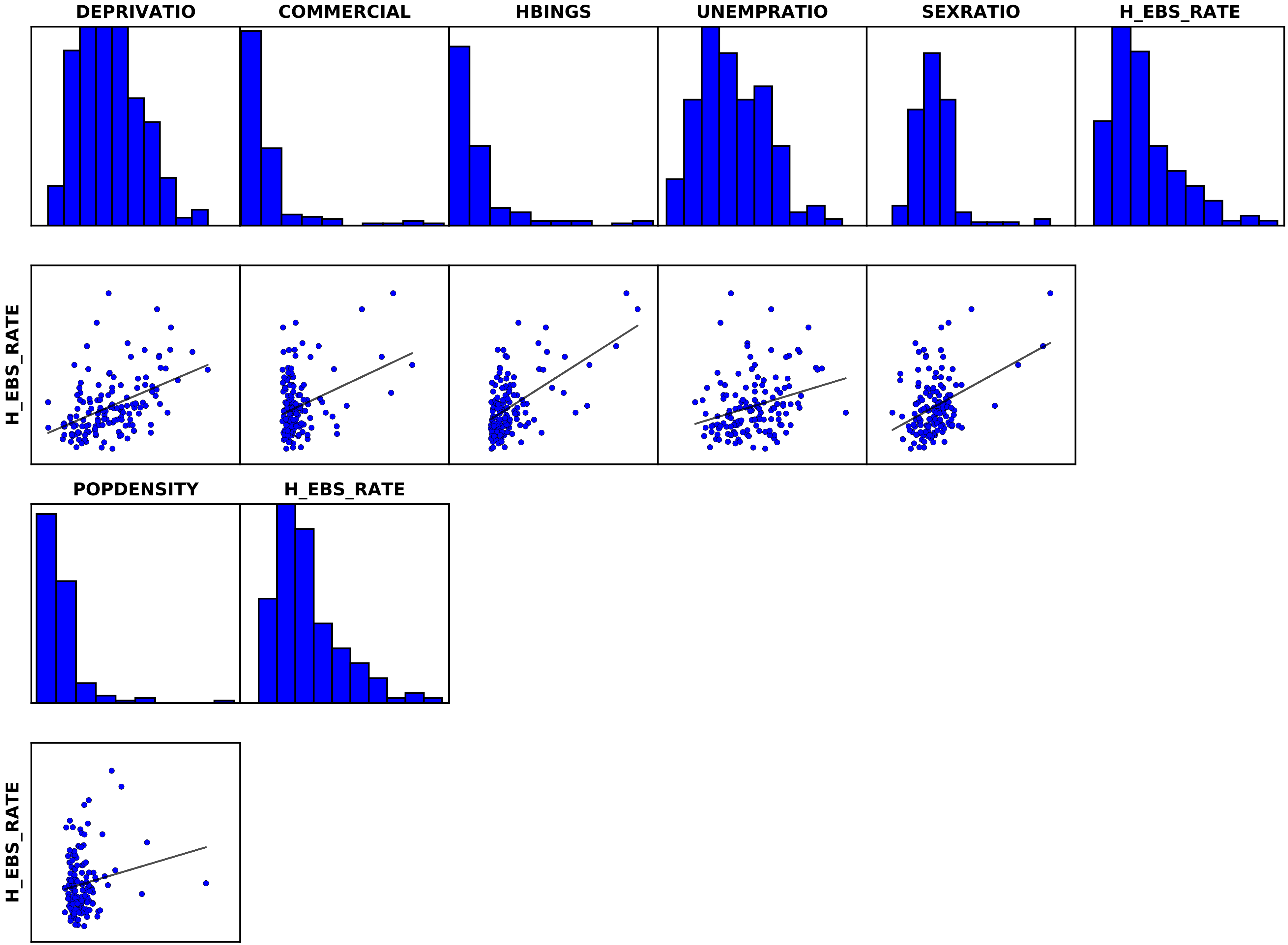

Variable Distributions and Relationships (Cont.)

The above graphs are Histograms and Scatterplots for each explanatory variable and the dependent variable. The histograms show how each variable is distributed. OLS does not require variables to be normally distributed. However, if you are having trouble finding a properly-specified model, you can try transforming strongly skewed variables to see if you get a better result.

Each scatterplot depicts the relationship between an explanatory variable and the dependent variable. Strong relationships appear as diagonals and the direction of the slant indicates if the relationship is positive or negative. Try transforming your variables if you detect any non-linear relationships. For more information see the Regression Analysis Basics documentation.

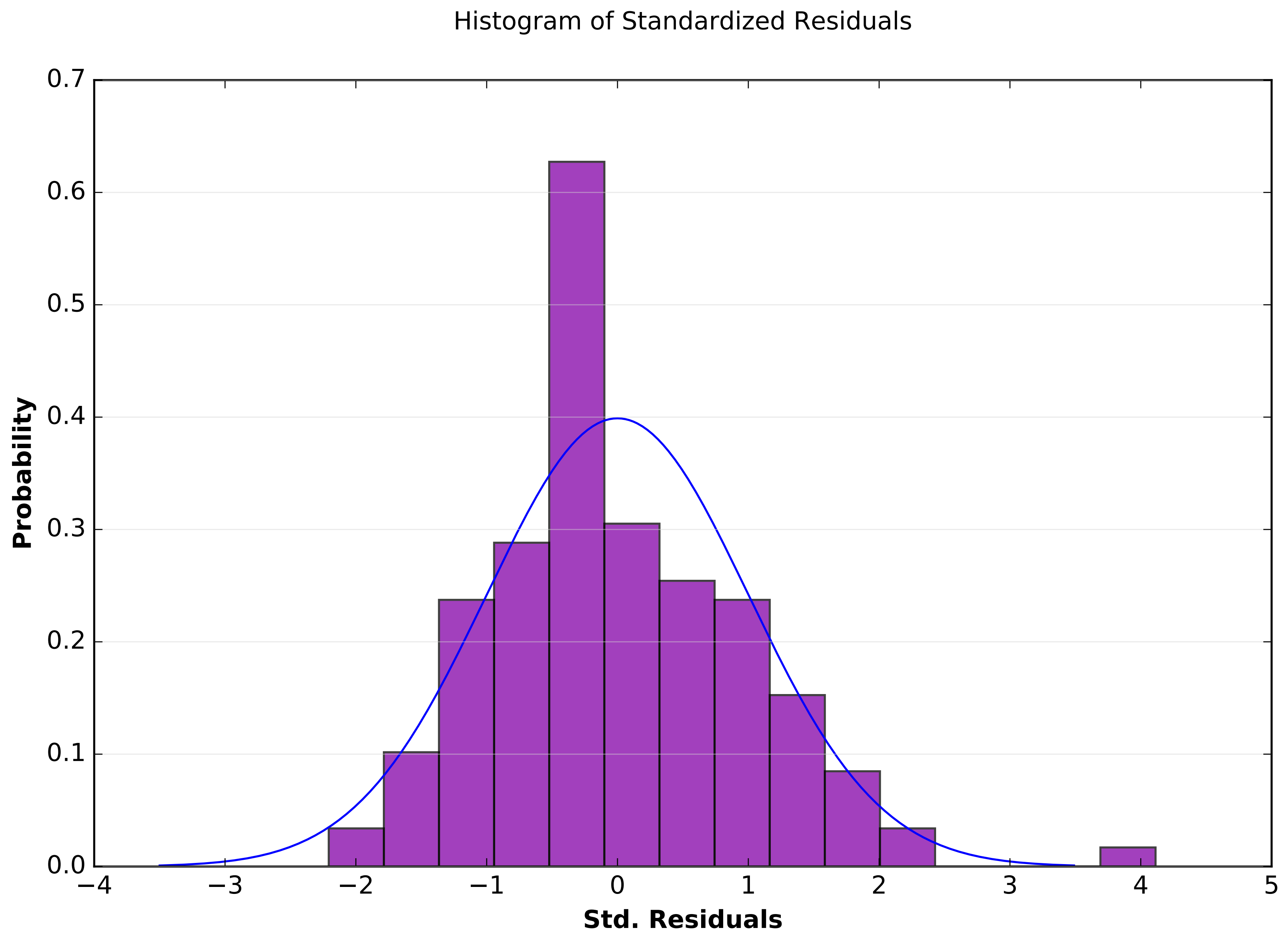

Ideally the histogram of your residuals would match the normal curve, indicated above in blue. If the histogram looks very different from the normal curve, you may have a biased model. If this bias is significant it will also be represented by a statistically significant Jarque-Bera p-value (*).

Residual vs. Predicted Plot

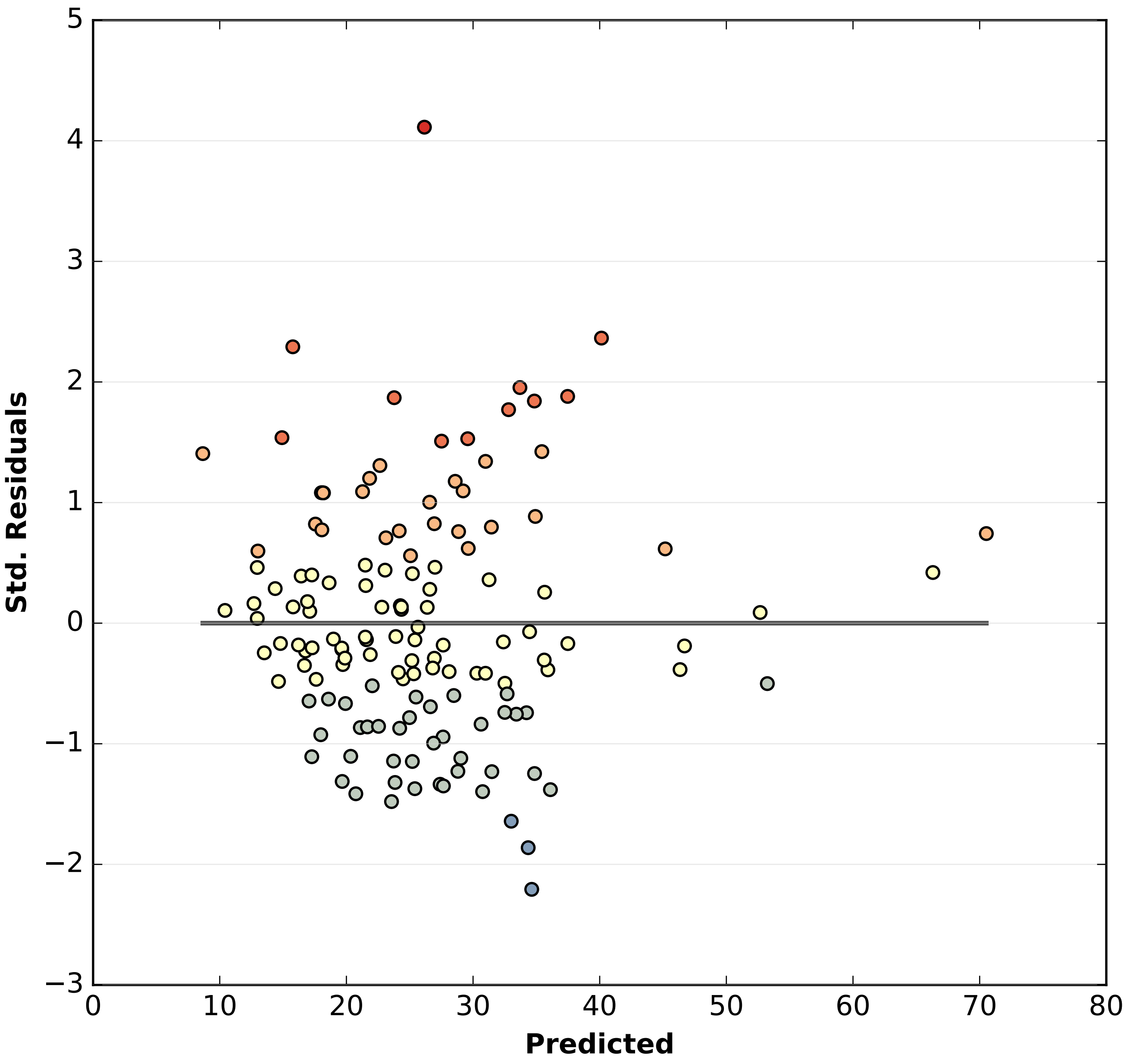

This is a graph of residuals (model over and under predictions) in relation to predicted dependent variable values. For a properly specified model, this scatterplot will have little structure, and look random (see graph on the right). If there is a structure to this plot, the type of structure may be a valuable clue to help you figure out what's going on.

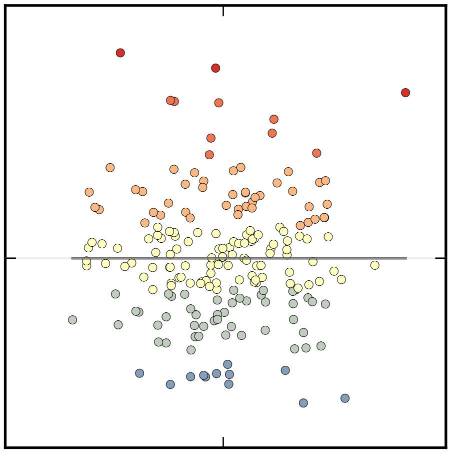

Random Residuals

Ordinary Least Squares Parameters

Parameter Name	Input Value
Input Features	Homicide
Unique ID Field	Hood_ID
Output Feature Class	None
Dependent Variable	H_EBS_RATE
Explanatory Variables	DEPRIVATIO
	COMMERCIAL
	HBINGS
	UNEMPRATIO
	SEXRATIO
	POPDENSITY
Selection Set	False
